# The Impact on Outcome by Adding Bevacizumab to Standard Induction Chemotherapy Prior to Mesothelioma Surgery: A Retrospective Single Center Analysis

**DOI:** 10.3389/fonc.2020.588563

**Published:** 2020-11-13

**Authors:** Olivia Lauk, Karina Bruestle, Thomas Neuer, Bianca Battilana, Thi Dan Linh Nguyen, Thomas Frauenfelder, Rolf Stahel, Walter Weder, Alessandra Curioni-Fontecedro, Isabelle Opitz

**Affiliations:** ^1^ Department of Thoracic Surgery, University Hospital Zurich, Zurich, Switzerland; ^2^ Institute of Diagnostic and Interventional Radiology, University Hospital Zurich, Zurich, Switzerland; ^3^ Department of Medical Oncology and Hematology, University Hospital Zurich, Zurich, Switzerland

**Keywords:** malignant pleural mesothelioma, anti-angiogenic therapy, bevacizumab, induction chemotherapy, macroscopic complete resection, pleurectomy/decortication

## Abstract

**Objectives:**

Adding bevacizumab, an anti-Vascular Endothelial Growth Factor (VEGF), to platinum-based chemotherapy/pemetrexed in 1^st^ line treatment of advanced malignant pleural mesothelioma (MPM), significantly improved overall survival. However, increased high grade bleeding after operation was reported in patients with colorectal cancer who previously received bevacizumab. In the present analysis, we assessed for the first time the impact of adding bevacizumab to induction chemotherapy prior to surgery for mesothelioma patients.

**Methods:**

Two hundred twenty-seven MPM patients, intended to be treated with induction chemotherapy followed by surgery at the University Hospital of Zurich between 2002 and December 2018, were included in the present analysis. After propensity score matching for gender, histology and age (1:3 ratio), data from 88 patients were analyzed. Sixty-six patients underwent induction chemotherapy (with cis-/carboplatin and pemetrexed: control group) alone and 22 patients underwent induction chemotherapy with the addition of bevacizumab (bevacizumab group) prior macroscopic complete resection (MCR). Perioperative and long-term outcome variables were analyzed.

**Results:**

Patients undergoing combination treatment with bevacizumab had a significantly better response than with chemotherapy alone as assessed by modified RECIST (p=0.046). Intraoperative complications in the bevacizumab group (one patient), or in the control group (three patients) were not related to intraoperative bleeding. Postoperative transfusion of blood products occurred in a larger amount in the control group than in the bevacizumab group (p=0.047). Overall survival was not statistically different between both groups.

**Conclusion:**

These initial data demonstrate that MCR can be performed safely after triple induction chemotherapy with bevacizumab without increased intra- and postoperative bleeding complications. Response rates were significantly improved by the addition of bevacizumab.

## Introduction

Malignant pleural mesothelioma (MPM) is an aggressive cancer with poor outcome despite multimodality treatment (1, 2). The Mesothelioma Avastin Cisplatin Pemetrexed Study (MAPS)—a multicenter, randomized, controlled, clinical phase III trial- showed the improvement of survival by the addition of bevacizumab to standard chemotherapy cisplatin/carboplatin/pemetrexed in first line treatment for advanced MPM (survival 18.8 months [95% CI 15.9-22.6] vs. 16.1 months [95% CI 14.0–17.9]) (3).

Vascular endothelial growth factor (VEGF) represents a target for cancer treatment as anti-VEGF agents can induce a direct toxicity by decreasing neoplastic cell viability. Moreover they induce structural changes on tumor vasculature with increase in stability, perfusion and permeability leading to an augmented distribution of chemotherapeutic agents at the tumor site (4). A previous meta-analysis of 20 randomized controlled trials reported an overall increase in high grade bleeding in colorectal, renal and non-small cell lung cancer, as well as an increase in thrombotic events (5–7) in patients undergoing treatment with anti-VEGF agents.

## Objectives

In the present analysis, we report for the first time, the perioperative and long-term outcome as well as chemotherapy response when bevacizumab is added to current standard platinum based induction chemotherapy for MPM patients undergoing subsequent macroscopic complete resection (MCR).

## Patients and Methods

This is an observational study of retrospective nature. The institutional database was searched for patients intended to be treated with induction chemotherapy followed by surgery (n=227) during the period 2002–2018. Some patients in both groups (bevacizumab and control group) were treated within our phase I and II trials of intracavitary application of cisplatin bound to a fibrin carrier (NCT01644994). Sixty-six patients were excluded due to missing data (n=35) or palliative therapy only (n=31). Propensity score matching (1:3 ratio), was performed in 161 patients based on age, gender, and histotype (see **Table 1**) between patients receiving induction chemotherapy (cisplatin/carboplatin/pemetrexed) with bevacizumab and induction chemotherapy (cisplatin/carboplatin/pemetrexed) (control group) followed by surgery. Follow-up was performed with computed tomography (CT) and positron emission tomography/computed tomography (PET/CT) scans in an alternating manner according to our institutional guidelines. This means a post-surgery quarterly clinical and radiological follow-up within the 1^st^ year, bi-annual in the 2^nd^ year, and from there on annually. A logistic regression model was fitted to obtain the propensity score with the application of bevacizumab as outcome and the three explanatory variables mentioned above to reduce potential sources of bias. The standardized mean difference was used to estimate the group differences, which is preferred over the sample size dependent t-test (see **Table 1**). The matching was performed without repetition of controls and a caliper distance within 0.2 times the standard deviation of the propensity scores was accepted as a match. For all cases obtaining bevacizumab three appropriate matches were found.

**Table 1:** Covariates before 1:3 matching (left) and after 1:3 matching (right).

Before 1:3 matching					After 1:3 matching				
Covariate	Overall	Control group	Bevacizumab group	SMD	Covariate	Overall	Control group	Bevacizumab group	SMD
**n**	**161**	**139**	**22**		**n**	**88**	**66**	**22**	
**Gender, male (%)**	139 (86.3)	121 (87.1)	18 (81.8)	0.145	**Gender, male (%)**	71 (80.7)	53 (80.3)	18 (81.8)	0.039
**Histotype, epithelioid (%)**	133 (82.6)	115 (82.7)	18 (81.8)	0.024	**Histotype, epithelioid (%)**	73 (83.0)	55 (83.3)	18 (81.8)	0.04
**Age** **(median [range])**	63 (33–76)	63 [33-76]	64.5 [50-76]	0.275	**Age** **(median [range])**	65 [40-76]	65 [40-76]	64.5 [50-76]	0.031

SMD, Standard mean difference. Sample size independent measure of group discrepancy.

For statistical analysis, R-software version 3.5.3 was used. Continuous variables were analyzed with paired t-test and conditional logistic regression was performed for binary variables. A p value of <0.05 was considered as statistically significant. Missing data are reflected in the overall column in each table. Throughout the manuscript, due to the missing adjustment for multiple testing in this analysis, p values should be interpreted as descriptive.

Local ethics committee approval was given for analysis of the mesothelioma database (StV 29-2009, EK-ZH 2012-0094).

### Staging and Induction Chemotherapy

Mesothelioma was diagnosed and staged as described previously (8). After completion of staging, patients received between two and seven cycles of induction chemotherapy either with cisplatin/carboplatin or carboplatin/pemetrexed with the addition of bevacizumab in 22 cases. In case of only two cycles applied, the chemotherapy was interrupted due to side effects and the patient proceeded to surgery. In case of seven cycles (n=1), the patient did not want to undergo surgery at first and decided at a later point to have surgery. The last cycle prior to surgery was conducted without bevacizumab. The decision for induction chemotherapy with the addition of bevacizumab was individually discussed for each patient at our interdisciplinary tumor-board based on the comorbidity profile of the patient (e.g. coagulation disorders).

### Surgery and Intraoperative Blood Loss

Thirty-one patients underwent (extended) pleurectomy/decortication [(E)PD), 35 extrapleural pneumonectomy (EPP)] in the control group whereas in the bevacizumab group 21 patients underwent (E)PD and one patient EPP. P/D only was performed in four patients in the control group and none in the bevacizumab group. EPP and (E)PD were performed as already described previously (8). The difference between EPD and P/D was that in EPD pericardium and/or diaphragm are additionally resected depending on the tumor spread. In uncertain cases this was decided under guidance of intraoperatively taken fresh frozen sections.

Blood loss during surgery as well as the amount of substituted erythrocyte concentrates were documented.

Further, hemoglobin, hematocrit and thrombocytes were measured on postoperative day (POD) 1 to 6; not all values were available for each day. Thirty- and 90-day mortality was reported as well as postoperative morbidity. Postoperative major morbidity included: complications necessitating reoperation, chylothorax, patch failure, empyema, bronchopleural fistula, thromboembolic events, and acute respiratory distress syndrome (ARDS). The number of thromboembolic events for each patient are reported in detail, in particular the time point of the events (before chemotherapy, after chemotherapy but before surgery, post-surgery and after adjuvant chemo-/radiotherapy).

### mRECIST

Chemotherapy response was assessed by mRECIST in a restaging CT scan (9). The p-value was calculated *via* a McNemar test.

### OS and PFS

Overall survival (OS) and progression free survival (PFS) probabilities were calculated with Kaplan Meier analysis and the difference between treatment groups was evaluated with a conditional long rank test. The median and 95% confidence interval were also determined using the Kaplan-Meier method. OS was calculated from start of induction chemotherapy until death or lost to follow up.

## Results

**Figure 1:**
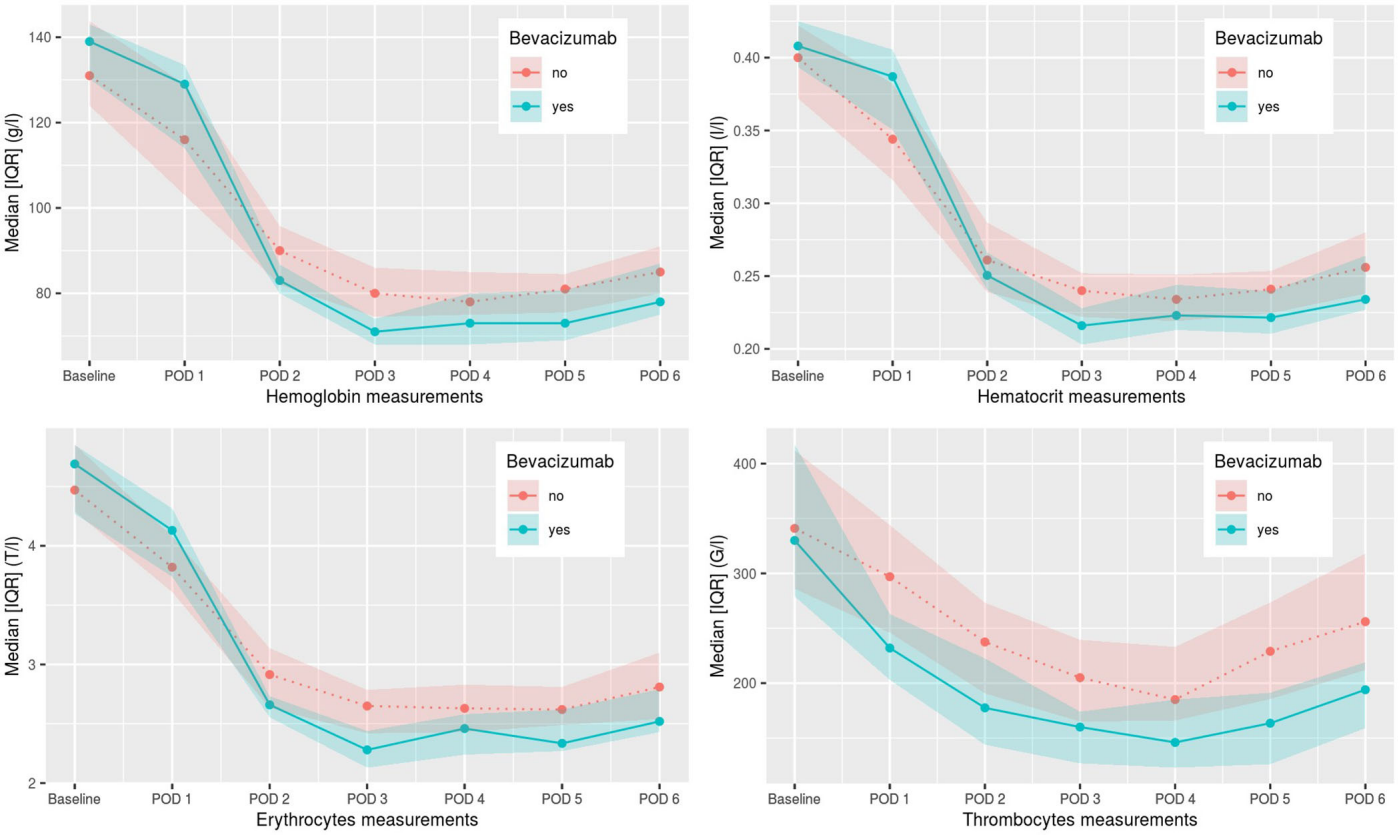
Postoperative hemoglobin, hematocrit, erythrocyte, and thrombocyte values from day 1–6. POD, postoperative day.

88 patients, receiving either induction chemotherapy (cisplatin/carboplatin/pemetrexed + bevacizumab) or induction chemotherapy (platinum based/pemetrexed) only, followed by surgery, were analyzed (**Figure 1**): 66 patients in the control group and 22 patients in the bevacizumab group [(see **Table 1**) after propensity score matching (1:3 ratio)]. Additionally, intracavitary chemotherapy was applied in nine patients of the control group and six patients of the bevacizumab group. Patient`s characteristics are shown in **Table 2**.

**Table 2:** Patients’ characteristics.

Covariate	Overall	Control group	Bevacizumab group	p-value
n	88	66	22	
**Age (median [range])**	65 [40-76]	65 [40-76]	64.5 [50-76]	NS
**Gender, male (%)**	71 (80.7)	53 (80.3)	18 (81.8)	NS
**Laterality of MPM, right (%)**	53 (60.2)	37 (56.1)	16 (72.7)	NS
**Epithelioid histotype (%)**	73 (83.0)	55 (83.3)	18 (81.8)	NS
**Induction chemotherapy**				
**Cisplatin (%)**	78 (88.6)	60 (90.9)	18 (81.8)	NS
**Carboplatin (%)**	18 (20.5)	11 (16.7)	7 (31.8)	NS
**Pemetrexed (%)**	87 (98.9)	65 (98.5)	22 (100.0)	NS
**Bevacizumab (%)**	22 (25.0)	0 (0.0)	22 (100.0)	**<0.001**
**Other (%)**	1 (1.1)	1 (1.5)	0 (0.0)	NS
**IMIG (%)**	12 (13.6)	5 (7.6)	7 (31.8)	NS
**IA**	17 (19.3)	14 (21.2)	3 (13.6)	
**IB**	37 (42.0)	28 (42.4)	9 (40.9)	
**II**	11 (12.5)	10 (15.2)	1 (4.5)	
**IIIA**	9 (10.2)	8 (12.1)	1 (4.5)	
**IIIB**	2 (2.3)	1 (1.5)	1 (4.5)	
**Type of surgery (%)**				**<0.001**
**EPD**	52 (59.1)	31 (47.91)	21 (95.5)	
**EPP**	36 (40.9)	35 (53.0)	1 (4.5)	
**P/D**	4 (4.5)	4 (6.1)	0 (0.0)	

IMIG, International Mesothelioma Interest Group, (E)PD, extended pleurectomy/decortication; EPP, extrapleural pneumonectomy; P/D, pleurectomy/decortication. Bold, statistically significant with a p-value <0.05.

The overall median age in this present cohort was 65 years (range 40–76 years) and did not differ significantly between both groups. All patients underwent surgery in a median time of 41.5 days (6 weeks, range 15–155 days) after the last cycle of induction chemotherapy. The median number of cycles applied for all patients was 3 (range 2–7). One patient had surgery <20 days following the last cycle of chemotherapy based on his own wish. Two patients underwent surgery after >100 days; one due to other surgery and the other based on patient’s wishes.

### mRECIST

According to mRECIST criteria (9) there were no patients with progressive disease (PD) in the bevacizumab group compared to 15 patients (22.7%) with PD in the control group following induction chemotherapy. Partial remission (PR) was observed in 23 cases in the control group (34.8%) and nine cases (40.9%) in the bevacizumab group. 28 cases (42.4%) in the control and 13 cases (59.1%) in the bevacizumab group, had stable disease (SD). The overall response rate was significantly better in the bevacizumab group with a p-value of 0.046.

Intraoperative complication rate and postoperative 30-day and 90-day mortality rate was not higher in the patient group undergoing induction chemotherapy with bevacizumab in comparison to the control group. Three patients in the control group and one patient in the bevacizumab group had intraoperative complications, none due to intraoperative bleeding. The number of postoperative transfusions of blood products was statistically different (p=0.047) in the two groups, with 68.2% of patients in the control group receiving postoperative blood products including mainly erythrocyte concentrates compared to 21 patients (95.5%) in the bevacizumab group (see **Table 3**). In few cases factor concentrate substitution, fresh frozen plasma, and thrombocyte concentrates were given as well. The postoperative hemoglobin, hematocrit, and thrombocyte values for both groups are shown in **Figure 1**. A statistically significant difference between the two groups was seen for hemoglobin values on POD 1-5, for hematocrit value on POD 5, for erythrocyte values on POD 2-6, for thrombocyte values on POD 1-6, and for hematocrit value on POD 1. For all these values, the bevacizumab group did better, except for the hematocrit value on POD 1. Additionally, no patient showed major pathological response and all patient still had microscopically vital tumor tissue in the definitive pathological tumor specimen.

**Table 3:** Surgery dependent variables.

Covariate	Overall	Control group	Bevacizumab group	p-value
n	88	66	22	
**Blood loss** **during surgery (ml, median [range])**	1000 [200–5000]	1000 [200–5000]	850 [400–3000]	NS
**Intraoperative blood products** **applied (pack) (%)**	32 (36.4)	26 (39.4)	6 (27.3)	NS
**Intraoperative ECs** **applied (ml, median [range])**	300 [0–1500]	600 [0–1500]	300 [300–600]	NS
**Intraoperative** **complications n (%)**	4 (4.5)	3 (4.5)	1 (4.5)	NS
**30 Day** **Mortality (%)**	2 (2.3)	1 (1.5)	1 (4.5)	NS
**90 Day** **Mortality (%)**	5 (5.7)	4 (6.1)	1 (4.5)	NS
**Postoperative hemorrhage** **necessitating reoperation (%)**	3 (3.4)	2 (3.0)	1 (4.5)	NS
**Postoperative** **morbidities (%)**	87 (98.9)	65 (98.5)	22 (100)	NS
**Postoperative** **transfusion of blood products (pack) (%)**	66 (75.0)	45 (68.2)	21 (95.5)	**0.047**

NS, Not statistically significant. Bold, statistically significant with a p-value <0.05.

Postoperative morbidity did not differ between the two groups with seven (32%) events in the bevacizumab group compared to 19 (29%) in the control group. Pulmonary embolism (PE) occurred in six patients in both groups (control 9.1% and bevacizumab group 27.3%; p=0.09). Three patients in each group had a thromboembolic event after induction therapy and one patient in each, for both groups, had a thromboembolic event either before chemotherapy started or after surgery, or after adjuvant chemo-/radiotherapy. There was no statistically significant difference in the occurrence of pulmonary embolism between the two groups in terms of time of occurrence and number of events. Occurrence of thromboembolic events according to the surgical procedure was as follow: EPP (n=1 for bevacizumab group vs n=1 for control group), EPD (n=5 vs. n=3), and P/D (n=0 vs. n=2).

Thirty-day mortality [control group n=1 (1.5%) and bevacizumab group n=1 (4.5%)] was not statistically different among the two groups as well as the 90-day mortality [control group n=4 (6.1%) and n=1 (4.5%, bevacizumab group)].

### OS and PFS

At the time point of analysis, 17 patients in the control group and nine patients in the bevacizumab group were still alive (two lost to follow up in the control group). Overall median follow up time was 41 months.

**Figure 2:**
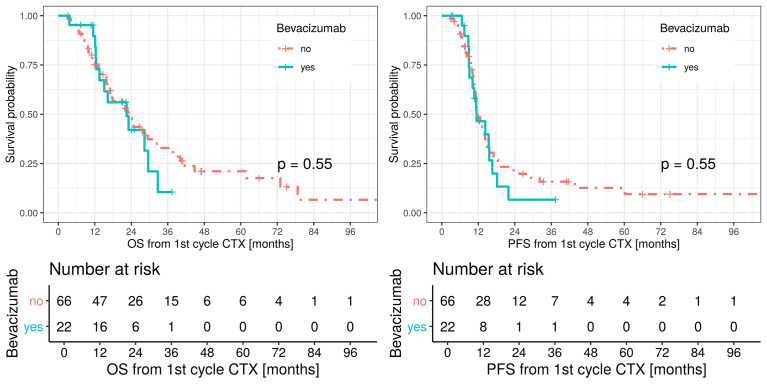
Overall survival (OS, left figure), red line patients treated with platinum-based/pemetrexed for induction chemotherapy and green line patients treated with triplet induction chemotherapy including bevacizumab. Median OS in the bevacizumab group 22.4 months (LCL 95%, 13.5, UCL 95%, not applicable). Median OS in the control group 23 months (LCL 95%, 17.3, UCL 95%, 33.5). Progression free survival (PFS, right figure), red line patients treated with platinum-based/pemetrexed for induction chemotherapy and green line patients treated with triplet induction chemotherapy including bevacizumab.

Median OS was 23 months (95% CI: 17.3–33.5) in patients undergoing induction chemotherapy only and 22.4 months in patients receiving induction chemotherapy and bevacizumab (95% CI: 13.5 – not applicable; p = 0.55) with no censored cases. Median PFS was 11.5 months (95% CI: 11.1–14.5) for the control group and 11.4 months (95% CI: 10.1–18.2) in the bevacizumab group. No statistical difference in the two groups was observed in terms of OS and PFS. OS and PFS are shown in **Figure 2**.

## Discussion

In this propensity score matched retrospective analysis, 22 patients with malignant pleural mesothelioma were treated with induction chemotherapy consisting of platinum-based/pem with bevacizumab prior to surgery compared to 66 patients receiving standard induction chemotherapy with platinum-based/pemetrexed alone. Whereas the concept of a multimodality treatment is widely accepted and recommended for this disease, the timing of chemotherapy before or after surgery is discussed controversially (1, 10, 11) and currently investigated in a multicenter randomized phase II trial by the EORTC [The European Organization for Research and Treatment of Cancer (NCT 02436733)].

In the present analysis, adding bevacizumab to standard induction chemotherapy did not significantly increase perioperative, in particular not bleeding, complications, although the proportional number of postoperative blood products and PE were higher compared to the control group. The small sample size and the lack of randomization do not allow to draw any conclusion to fully answer this question as well as the fact that PE is in general a known side effect of also the other chemotherapeutic agents. As known in other settings, these data suggest that a careful monitoring of embolic events in patients undergoing bevacizumab is warranted.

Bevacizumab is a well-known and potent VEGF inhibitor and has been mostly used as an additional chemotherapeutic agent, especially, in solid tumors including malignant mesothelioma, non-small cell lung cancer, colorectal cancer, renal cancer, and breast cancer. In malignant pleural mesothelioma patients, anti-VEGF has already been investigated in several studies for its safety and efficacy (7, 12). One of the main side effects is an increased risk of bleeding ranging from minor epistaxis to fatal hemorrhage (7, 13).

Despite evidence of an increased bleeding risk with bevacizumab, surgery was demonstrated to be safe in our cohort with negligible intra- and postoperative bleeding complications and even less intraoperative blood loss (750 ml vs. 900 ml) in the bevacizumab group, if a time interval of 2–7 weeks after induction chemotherapy, and the exclusion of bevacizumab in the last cycle prior to surgery, was respected. By recommendation of the Food and Drug Administration (FDA), bevacizumab should not be administered less than 28 days before and after a surgical intervention (14).

Numerical wise, in the bevacizumab group, more postoperative bleeding complications occurred although without statistical significance compared to the control group. Two cases in the control group necessitated reoperation due to hemorrhage after EPP and one case in the bevacizumab group. One hypothesis might be of a greater postoperative bleeding risk after EPP compared to (E)PD (8, 15, 16), but the risk for intra- or postoperative bleeding complications arise from the big bleeding surface after parietal pleurectomy part (being part of both procedures) (8).

Thirty-day- and 90-day mortality in the bevacizumab group was very low with one case each compared to the control group with one and four cases, respectively. The reasons for postoperative mortality were postoperative empyema, patch failure with gastric herniation, global respiratory insufficiency after EPP and necrotizing pancreatitis most probably related to the surgical procedure itself.

Due to the long observation period, there have been more EPP performed in the control group than in the (E)PD group. The learning curve for a better patient selection, the procedures and complication management might also play a significant role for the minor postoperative bleeding complication in the EPD/bevacizumab group. Additionally, preoperative selection criteria improved over the past years. On restaging imaging by contrast enhanced CT scan or even PET/CT after induction chemotherapy, there is a routine assessment of mRECIST, whereby patients are classified to their tumor response to induction chemotherapy.

A marginal statistically significant difference in terms of a better response was seen for the bevacizumab group. This improved response did not translate into a survival benefit.

There have been controversial debates on the efficacy and overall benefit of bevacizumab treatment, both for single agent use and for additive use (17–19). Several studies failed to show a clear benefit in OS or PFS when adding bevacizumab to standard therapy regimens for patients with malignant pleural mesothelioma. This may be due to the small sample sizes in the cohorts (17–19). This holds true for our analysis, as PFS and OS in this cohort did not show a statistically significant improvement when bevacizumab was added to standard chemotherapy.

Our results are in line with the first randomized double blind placebo-controlled phase II trial by Kindler et al. where bevacizumab was added to gemcitabine and cisplatin and both PFS and OS did not differ statistically significant [PFS of 6.9 months compared to 6.0 months without bevacizumab (p=0.88) and an OS of 15.6 months compared to 14.7 months, respectively (p=0.91)] (17).

However, the MAPS trial, the first phase III trial investigated the addition of bevacizumab to standard chemotherapy regime proofed the beneficial effect of bevacizumab as the primary outcome of OS was significantly extended [median OS 18.8 months vs. 16.1 months without bevacizumab (p=0.0167)] (3).

We are aware of the limitations of this study mostly related to the retrospective nature, even though we performed a 1:3 propensity score match. Further stratification for more homogenous distribution of patients in each treatment group was not applicable due to the limited number of patients treated with additional bevacizumab and next to the rarity of this disease and the novel and not yet standardized use of bevacizumab. The outcomes overall survival, progression free survival, postoperative morbidity and mortality as well as response to chemotherapy given as mRECIST may be influenced by potential cofounders in this propensity score matched analysis. Due to the retrospective nature of this analysis, there might have been an improvement in patient management over the time, especially regarding the general learning curve and a transition from EPP to (E)PD, on top of the lower mortality rate in the latter group per se, in parallel to the patient allocation to bevacizumab in the later period.

The missing impact on overall survival additionally may be due to this antiangiogenic drug being newly used as an addition to standard induction chemotherapy (17–19). Secondly, the bevacizumab group was the one with more patients still alive, this was in the majority given to the fact, that those were the more recently treated patients. The scarce statistically significant difference in terms of response was seen in the bevacizumab group, as well as the missing benefit of overall survival underlies again that these results must been taken with caution due to the small sample size.

Despite all these new therapy approaches further trials are warranted to find an ideal therapy regimen balancing toxicity and efficacy. Although, increased bleeding risk with bevacizumab is a known common side effect, mesothelioma surgery can still be performed safely if appropriate time intervals are respected. We conclude, this therapy approach, including bevacizumab, warrants further investigation into a larger number of patients in order to finally characterize safety and outcome for patients with mesothelioma.

## Meeting Presentation

This work was presented at the 27^th^ ESTS annual meeting in Dublin, Ireland on Tuesday 11^th^ of June in the Chest Wall/Diaphragm/Pleura Session XIII (2pm–3pm).

## Data Availability Statement

The raw data supporting the conclusions of this article will be made available by the authors, without undue reservation.

## Ethics Statement

Local ethics committee approval was given for analysis of the mesothelioma database (StV 29-2009, EK-ZH 2012-0094). The patients/participants provided their written informed consent to have their anonymized data collected.

## Author Contributions

OL: Creation and presentation of the published work, specifically writing the initial draft (including substantive translation), Conceived and designed the analysis. KB: Drafted and provided critical revision of the article. TN: Performed the analysis, Conceived and designed the analysis. BB: Collected the data. TDLN: Drafted and provided critical revision of the article. TF: Drafted and provided critical revision of the article. AC-F: Drafted and provided critical revision of the article. RS: Drafted and provided critical revision of the article. WW: Drafted and provided critical revision of the article. IO: Oversight and leadership responsibility for the research activity planning and execution, including mentorship external to the core team. All authors contributed to the article and approved the submitted version.

## Funding

This work was partially funded by the Swiss National Science Foundation (PP00P3_133657 and PP00P3_159269).

## Conflict of Interest

The authors declare that the research was conducted in the absence of any commercial or financial relationships that could be construed as a potential conflict of interest.
